# Simultaneous and sequential based co-fermentations of *Trichoderma asperellum* GDFS1009 and *Bacillus amyloliquefaciens* 1841: a strategy to enhance the gene expression and metabolites to improve the bio-control and plant growth promoting activity

**DOI:** 10.1186/s12934-019-1233-7

**Published:** 2019-10-29

**Authors:** Valliappan Karuppiah, Murugappan Vallikkannu, Tingting Li, Jie Chen

**Affiliations:** 1 School of Agriculture and Biology, Shanghai Jiao Tong University, Shanghai, People’s Republic of China; 2 The State Key Laboratory of Microbial Metabolism, Shanghai Jiao Tong University, Shanghai, People’s Republic of China

**Keywords:** Simultaneous inoculation, Sequential inoculation, Co-cultivation, *T. asperellum*, *B.* *amyloliquefaciens*, Metabolomics, Plant growth, Biocontrol

## Abstract

**Background:**

The consequence of simultaneous and sequential inoculation of *T. asperellum* and *B. amyloliquefaciens* cultures with respect to growth rate, differential expression of vital genes and metabolites were examined.

**Results:**

The competition was observed between *T. asperellum* and *B. amyloliquefaciens* under co-cultivation. The proliferation of *Trichoderma* was reduced in the simultaneous inoculation (TB1) method, possibly due to the fastest growth of *Bacillus*. Both *T. asperellum* and *B. amyloliquefaciens* were proliferated in sequential inoculation method (TB2). The sequential inoculation method (TB2) upregulated the expression of metabolites and vital genes (sporulation, secondary metabolites, mycoparasitism enzymes and antioxidants) in *Trichoderma* and downregulated in *Bacillus* and vice versa in co-inoculation method (TB1). The metabolic changes in the co-culture promoted the maize plant growth and defense potential under normal and biotic stress conditions.

**Conclusion:**

The metabolites produced by the co-culture of *T. asperellum* and *B. amyloliquefaciens* improved the maize plant growth and defense potential under normal and biotic stress conditions.

## Background

Plant growth-promoting and biocontrol microorganisms survive together in the soil and use various strategies to enhance the plant growth and defense potential [1]. Considering the microbial living together strategy, blending of different microbial communities were developed to fulfill the agricultural needs [2]. Application of microbial consortium on plants were considered as an advanced method to control the infections. Further, microbial consortium increased the efficiency and stability of the microbes in different soil and ecological conditions [2]. The microbes such as *Trichoderma*, *Bacillus*, *Pseudomonas*, *Rhizobium*, *Glomus*, etc., were utilized to develop the microbial consortia [3]. Jain et al. [3] designed a viable microbial consortia to upregulate the plant defense genes. While, co-inoculation of *Azospirillum* and *Pseudomonas fluorescens* on cotton increased the yield [4].

The advancement of microbial fertilizer involved the development of microbial consortia by mixing two independent microbial culture [2, 5]. In this way, we aimed to find an optimal condition for co-culturing the two distinctive microbes and to identify their efficacy on plant growth and bio-control activity. In addition, Co-cultivation has turned into a specific inducer of activating the silent genes through the intercommunication and competition between each microbe [6].

The genus *Trichoderma* is a filamentous soil and plant root associated fungi. It is one of the important biocontrol species, demonstrating over 60% of all the listed biocontrol agents (BCA) used to reduce the plant infectious diseases [7–9]. The mechanism behind the plant growth and disease control by *Trichoderma* involves (1) the secretion of cell wall hydrolytic enzymes; (2) conversion of large substrates into the smaller and available forms to be utilized by plants; (3) holding an extraordinary resistance against the chemical fungicides; (4) reduce the amount of pathogens surrounding the plant roots by making the competition for nutrients and space; (5) inhibition of pathogen growth by mycoparasitism and secondary metabolite production; (6) increase the antioxidant and systemic resistance of plants; (7) induce the plant growth by the secretion of plant growth promoting molecules [10–12].

Previous research has been reported that *B. amyloliquefaciens* is an effective BCA to fight against the different range of plant pathogens [13, 14]. The mechanism likely involves the production of various antimicrobial compounds, competition for nutrients and space, and increase the plant resistance [15, 16]. In addition, disease reduction has been attributed by promoting the growth of plant beneficial micro-organisms in the rhizosphere, thereby it inhibit the growth and survival of pathogens and increase the soil enzyme activity [14, 17–19].

The application of multiple microbes is a simple way to improve the biocontrol effects [5]. However, in most cases, the simple mixing of *Trichoderma* and *Bacillus* at a certain ratio were used as the biocontrol agents. While, the co-cultivation of two distinctive microbes for agricultural applications were less studied. Previously, the co-culture of *Trichodema* and *Bacillus* were studied to understand the changes in the metabolism and gene expression [20, 21]. Metabolomics study confirmed the stimulation of different metabolites in co-inoculation based co-culture of *Trichodema* and *Bacillus* [20]. Karuppiah et al. [21] reported that sequential inoculation based co-culture had induced the differential gene expression of vital genes and secondary metabolites to enhance the antagonistic and bio-control activity. These studies did not explore the impact of inoculation time of *Trichoderma* and *Bacillus* on the differential expression of metabolites and genes under the co-culture conditions. Since the inoculation sequence is essential for growth of both microbes and production of agriculturally important metabolites, the impact of simultaneous and sequential inoculation of *Bacillus amyloliquefaciens* 1841 into the *Trichoderma asperellum* GDFS1009 were explored to understand the interaction, differential expression of vital genes and metabolites on the plant growth and biocontrol potential.

## Results

### Co-cultivation of *T. asperellum* GDFS1009 and *B. amyloliquefaciens* 1841

In the present investigation, the growth of *B. amyloliquefaciens* 1841 and *T. asperellum* GDFS1009 was evaluated using co-inoculation and sequential inoculation based techniques. After fermentation, the axenic culture of *B. amyloliquefaciens* in YMC medium was found to be creamy yellow with 3.7 × 10^11^ CFU/ml. The color of the YMC medium inoculated with the axenic culture of *T. asperellum* GDFS1009 (8 × 10^8^) was changed into tremendously lighter and beige with an even dissemination of mycelia with low viscosity. The co-inoculation of *B. amyloliquefaciens* ACCC11060 and *T. asperellum* GDFS1009 changed the medium into creamy yellow with high viscosity. The co-inoculation was dominated with *Bacillus* (5 × 10^11^) and limited *T. asperellum* GDFS1009 (8 × 10^3^) mycelia. In sequential inoculation method, the *T. asperellum* (4 × 10^8^) was enriched along with the *B. amyloliquefaciens* (3 × 10^10^). The fermentation medium of TB2 became creamy yellow after inoculation of *Bacillus*.

### Effect of sequential and co-inoculation on the Differential Gene Expression of *T. asperellum* and *B. amyloliquefaciens*

Distinct molecular expressions were observed between sequential and co-inoculation based co-cultivation of *T. asperellum* and *B. amyloliquefaciens*. The differences in the fold of gene expression under co-cultivations were evaluated using qRT-PCR with reference to their axenic controls. The cQ value of each gene category were normalized with 18S rRNA and 16S rRNA genes for *T. asperellum* and *B. amyloliquefaciens*, respectively. The analysis of the transcriptional response of *T. asperellum* under co-cultivation revealed that sequential inoculation upregulated the expression of genes related to the sporulation, secondary metabolism, antagonism and plant growth promoting related enzymes and antioxidants, while these genes were downregulated in co-inoculation based co-culture. The genes such as velvet (*Vel* 1), mitogen-activated protein kinase (*TMKa*), G protein receptor 1 (*GPR1*), blue-light-regulated genes (*BLR1* and *BLR2*) involved in the *Trichoderma* sporulation were induced in the sequential inoculation based co-cultivation. Similarly, non-ribosomal peptide synthetase (*NP1* and *NP2*), Putative ferrichrome synthetase (*NP3*), Cytochrome P450 (*Tri* 13) 1,*O*-methyl transferase (*OMT*) and Polyketide synthetase (*PK1* and *PK2*) were induced in the sequential inoculation based co-culture.

**Fig. 1:**
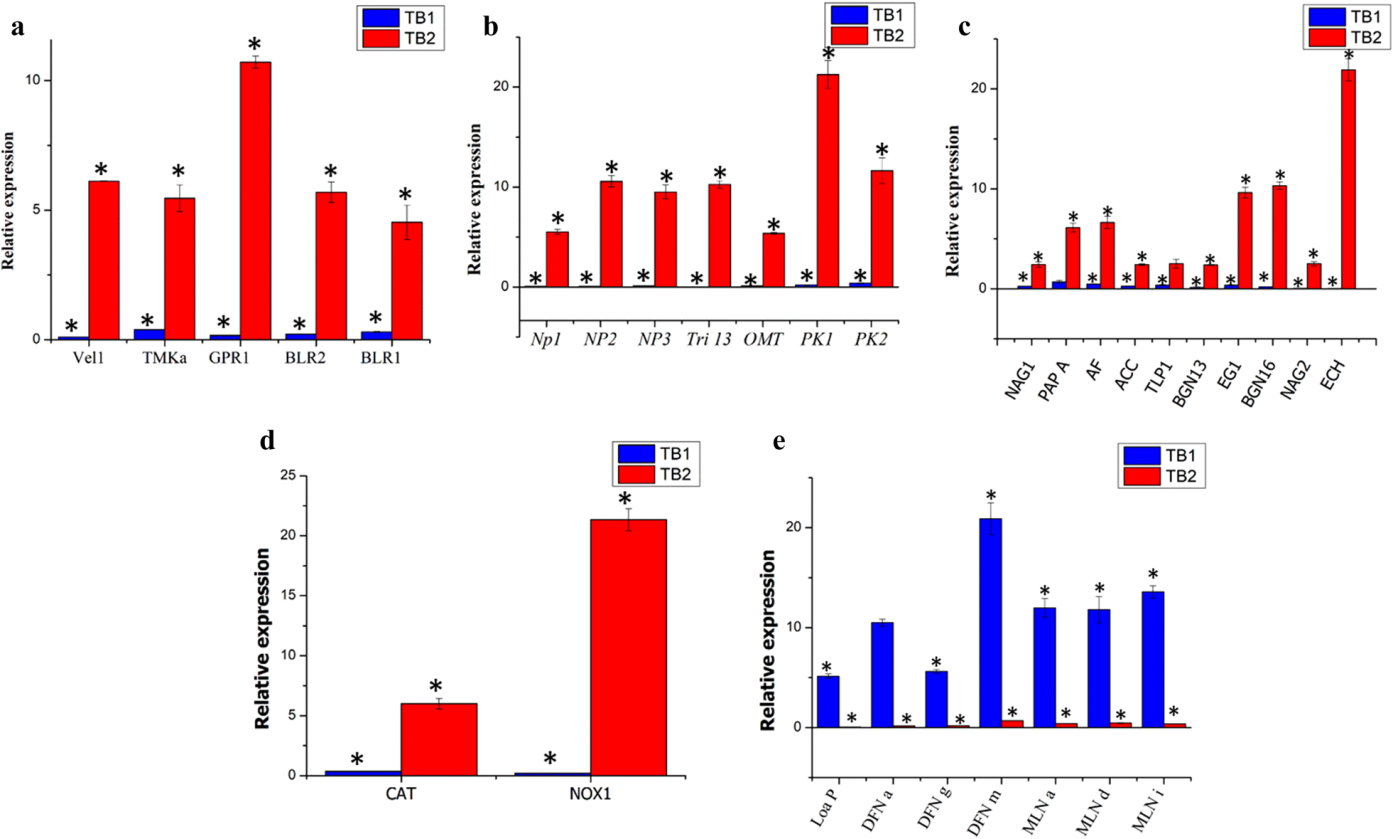
Influence of simultaneous (TB1) and sequential (TB2) inoculation based co-cultivation on the gene expression of (**a**) the *T. asperellum* morphology related genes [Velvet (*Vel1*), mitogen-activated protein Kinase (*TMKa*), G protein receptor 1 (*GPR1*), blue-light-regulated genes (*BLR1* and BLR2) and ENVOY (ENV1)], (**b**) secondary metabolism related genes [non-ribosomal peptide synthetase (*NP1* and *NP2*), Putative ferrichrome synthetase (*NP3*), Cytochrome P450 (*Tri* 13) 1,*O*-methyl transferase (*OMT*) and Polyketide synthetase (PK1 and PK2)]; (**c**) mycoparasitism-related genes [chitinase (ECH), β-1,3-glucanase (*BGN13*), β-1,6-glucanase (*BGN16*), β-1,4-glucanase (*Egl*), *N*-acetyl-glucosaminidases (*NAG1* and *NAG2*), aspartyl protease (*PAP A*), trypsin-like protease (*TLP 1*) and α-l-arabinofuranosidases (*AF*)]; and plant growth promoting enzyme [1-Aminocyclopropane-1-carboxylate deaminase (*ACC*)]. **d** Anti-oxidant genes [NADPH oxidase (*NOX*), catalase (*CAT*)] and (**e**) genes encoding *B. amyloliquefaciens* macrolactin and difficidin in the co-culture [intrinsic terminators located within the polyketide synthase (*PKS*) gene cluster encoding for the antibiotic difficidin (*DFN*) (*Loa P*), beginning, middle and end of the difficidin operon (*DFN A, DFN G,* and *DFN M*); beginning, middle and end of the macrolactin operon (*MLN a, MLN d,* and *MLN i*)]. Bars represent the standard error of the mean values of three biological replicates. ∗ represent significant differences between the axenic and co-culture (P < 0.05)

The expression of genes involved in the mycoparasitism were estimated in both axenic (T and B) and co-culture (TB1 and TB2). The expression of chitinase (*Ech*) was upregulated 21.9 fold in sequential inoculation based co-cultivation, whereas it was downregulated (0.038578 fold) in co-inoculation technique (Fig. 1). Similarly, the expression of β-1,3-glucanase (*BGN13*), β-1,6-glucanase (*BGN16*), β-1,4-glucanase (*Egl*), *N*-acetyl-glucosaminidases (*NAG1* and *NAG2*), aspartyl protease (*PAP* A), trypsin-like protease (*TLP* 1) and 1-aminocyclopropane-1-carboxylate deaminase (*ACC*) in sequential inoculation method was increased 0.15, 0.2, 0.3, 0.05, 0.2, 0.7, 0.35 and 0.26 folds, respectively, as compared to the axenic culture. The genes involved in the antioxidant such as NADPH oxidases (*NOX*) and catalase (*CAT*) were up-regulated 21.3, and 5.9 folds in the TB2. As shown in Fig. 1, 2^−ΔΔCT^ of the genes involved in growth, secondary metabolites, mycoparasitism and plant growth promoting related enzymes and antioxidants in TB1 were lesser than 1, it indicates that expression of some *Trichoderma* genes were down-regulated in TB1.

The production of bioactive substances by *B. amyloliquefaciens* under co-cultivation was analyzed by the expression of macrolactin (*MLN a*, *MLN d* and *MLN i*) and difficidin genes *(DFN a*, *DFN g* and *DFN m*). Figure 1 shows that co-inoculation based co-cultivation (TB1) significantly increased the expression of macrolactin and difficidin genes, while sequential inoculation (TB2) technique significantly reduced the expression of macrolactin and difficidin genes.

### Enzyme assay

**Fig. 2:**
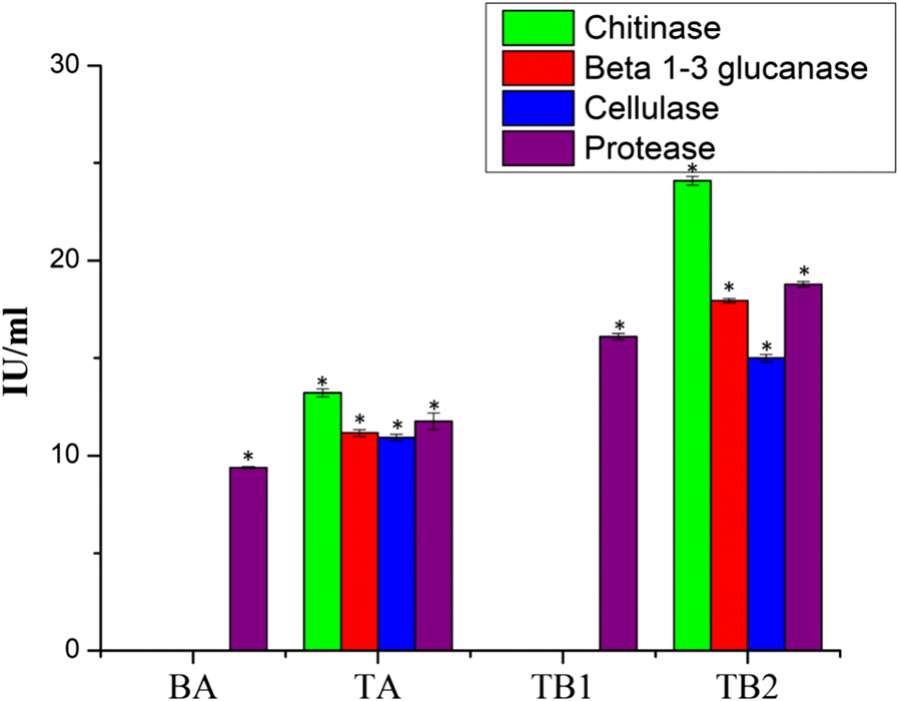
Enzyme activities associated with culture filtrates from the axenic (T and B) and co-culture (TB1 and TB2) of *T. asperellum* and *B. amyloliquefaciens* grown on YMC medium. (BA) axenic culture of *B. amyloliquefaciens* (TA) axenic culture of *T. asperellum* (TB1) simultaneous inoculation based co-culture (TB2) sequential inoculation based co-culture. Bars represent the standard error of the mean values of five biological replicates. ∗ represent significant differences between the axenic and co-culture (P < 0.05)

In order to examine differences in the mycoparasitism related enzyme activity in the co-culture, the enzymes such as *β*-1,3 glucanase, chitinase, cellulase and protease were accessed in the axenic (T and B) and co-culture (TB1 and TB2) (Fig. 2). The activities of *β*-1,3 glucanase, chitinase, cellulase and protease in the TB2 were elevated to 17.9, 24.1, 15, and 18.7 IU/mL, respectively. The increment of enzyme activity in TB2 was concurred with the upregulation of mycoparasitism related gene expression. Protease activity of TB1 was increased to 16.1 IU/mL, while the other enzyme activity such as *β*-1,3 glucanase, chitinase, and cellulase were not detected in the TB1 and B. The enzyme activities detected in the TB1 and TB2 were higher than the axenic cultures (Fig. 2).

### Metabolic profiles of axenic and co-culture

**Fig. 3:**
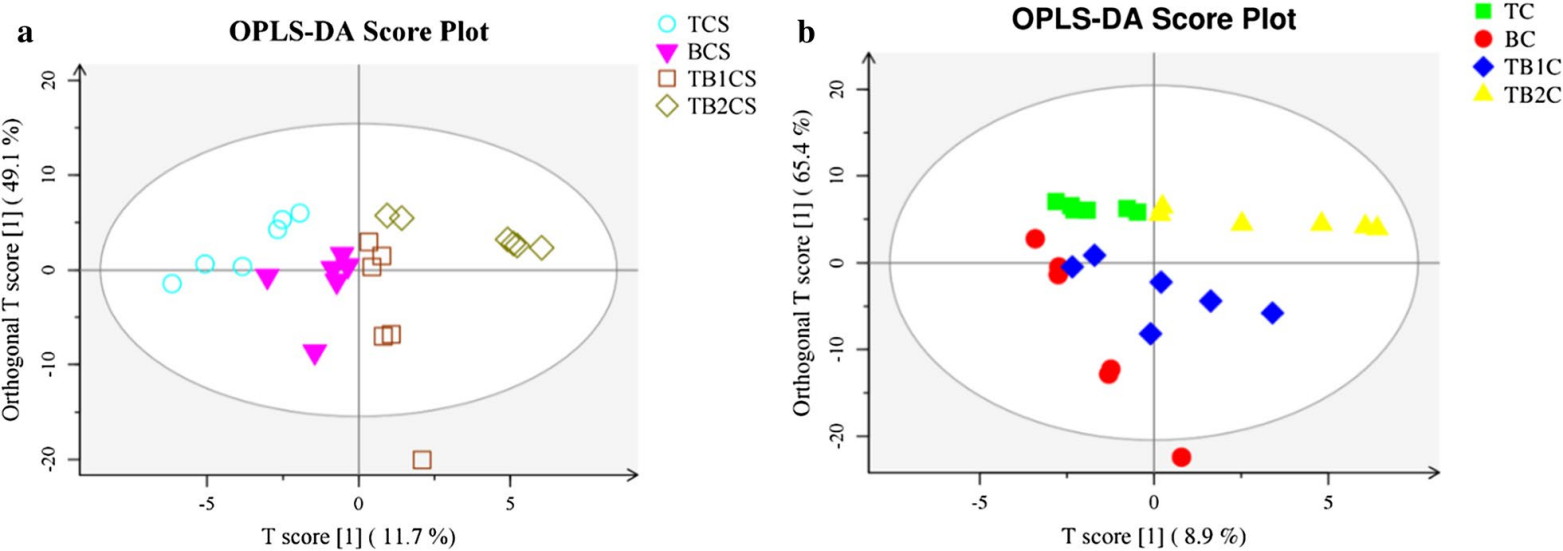
OPLS-DA analysis of the metabolic differences between the axenic (T and B) and co-culture (TB1 and TB2) of and *T. asperellum* and B. *amyloliquefaciens* based on LC–MS. **a** Extracellular based metabolic differences. **b** Intracellular based metabolic differences. (TCS) Culture supernatant of *T. asperellum* axenic culture (BCS) Culture supernatant of *B. amyloliquefaciens* axenic culture (TB1CS) Culture supernatant of simultaneous inoculation based co-culture (TB2CS) Culture supernatant of sequential inoculation based co-culture (TC) Culture pellet of *T. asperellum* axenic culture (BC) Culture pellet of *B. amyloliquefaciens* axenic culture (TB1C) Culture pellet of simultaneous inoculation based co-culture (TB2C) Culture pellet of sequential inoculation based co-culture

The modifications in the metabolic patterns of the co-culture and axenic culture were studied using the LC–MS technique. The OPLS-DA investigation of metabolite contents in both extracellular and intracellular showed the significant differences between the metabolites of axenic (T and B) and co-culture (TB1 and TB2) (Fig. 3a, b). This results was reliable with principal component analysis (Additional file 1: Fig. S1).

**Fig. 4:**
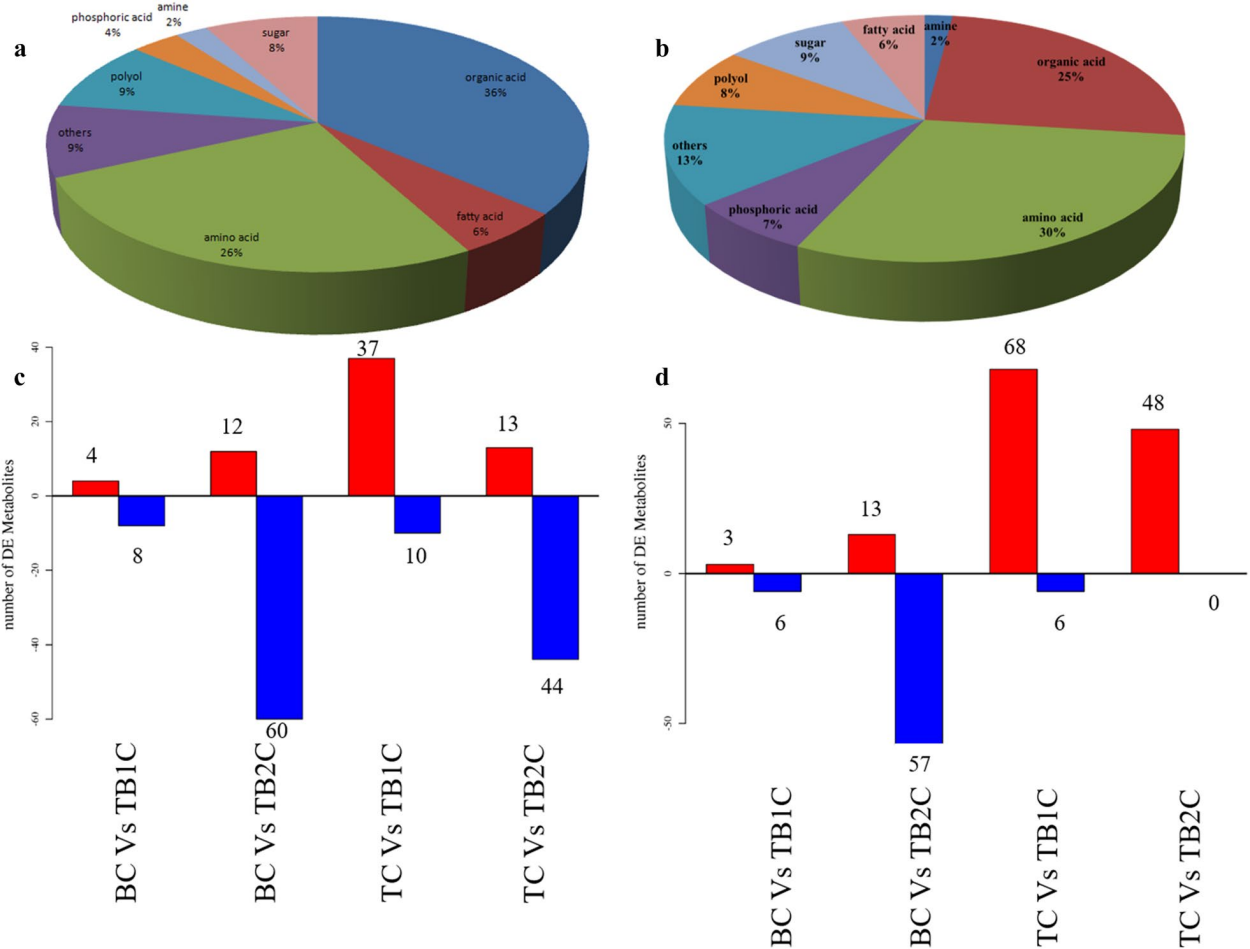
Classification and regulation of the metabolites in both extracellular and intracellular level of axenic and co-culture of and *T. asperellum* and B. *amyloliquefaciens* based on LC–MS. **a** Extracellular metabolic classification, **b** Intracellular metabolic classification, **c** extracellular metabolic regulation between the axenic and co-culture (**d**) intracellular metabolic regulation between the axenic and co-culture. (TCS) Culture supernatant of *T. asperellum* axenic culture (BCS) Culture supernatant of *B. amyloliquefaciens* axenic culture (TB1CS) Culture supernatant of simultaneous inoculation based co-culture (TB2CS) Culture supernatant of sequential inoculation based co-culture (TC) Culture pellet of *T. asperellum* axenic culture (BC) Culture pellet of *B. amyloliquefaciens* axenic culture (TB1C) Culture pellet of simultaneous inoculation based co-culture (TB2C) Culture pellet of sequential inoculation based co-culture

Hierarchical cluster analysis was carried out to assess the level of metabolites changes between the axenic (T and B) and co-culture (TB1 and TB2). This method explained variations between metabolic processes of each group (Additional file 1: Fig. S2). Totally, 101 and 89 metabolites were detected in the intracellular and extracellular level, respectively. These metabolites include amino acids, organic acids, carbohydrates, fatty acids, polyol, phosphoric acid, amines and others (Fig. 4). In extracellular, 37 and 4 metabolites from TB1 were upregulated compared to the T and B, respectively. While, 12 and 13 metabolites of TB2 were up regulated compared to the T and B, respectively. In contrast, 68 and 48 intracellular metabolites of TB1 and TB2 were up regulated compared to the T. 3 and 13 metabolites of TB1 and TB2 were upregulated compared to the B. In TB1, 6 metabolites were down regulated when compared to the axenic culture. None of the metabolites of TB2 were downregulated when compared to the T.

**Fig. 5:**
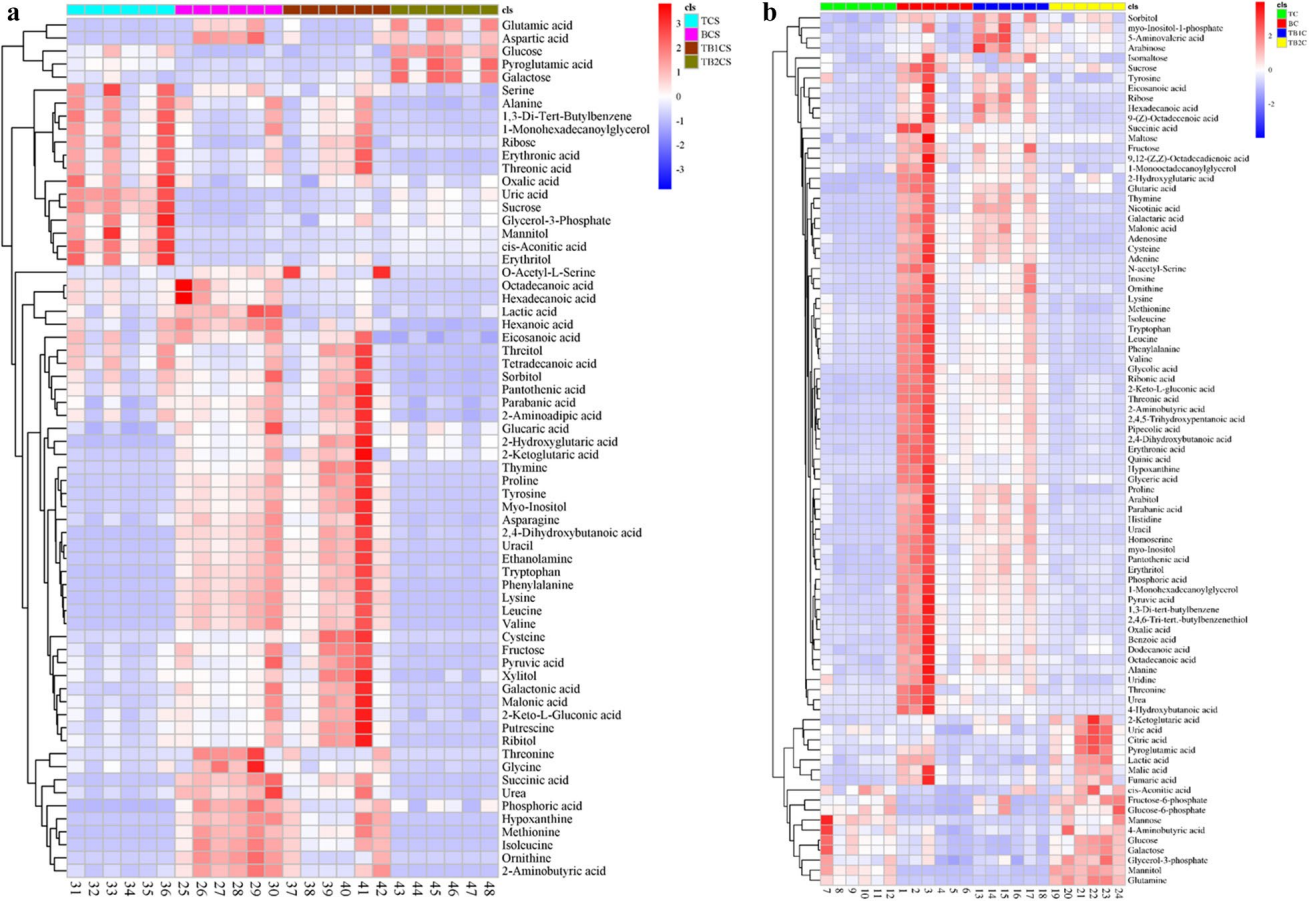
Metabolic differences between the axenic (T and B) and co-culture (TB1 and TB2) of and *T. asperellum* and B. *amyloliquefaciens* based on LC–MS. **a** Heat map of extracellular metabolites. **b** Heat map of intracellular metabolites. (TCS) Culture supernatant of *T. asperellum* axenic culture (BCS) Culture supernatant of *B. amyloliquefaciens* axenic culture (TB1CS) Culture supernatant of simultaneous inoculation based co-culture (TB2CS) Culture supernatant of sequential inoculation based co-culture (TC) Culture pellet of *T. asperellum* axenic culture (BC) Culture pellet of *B. amyloliquefaciens* axenic culture (TB1C) Culture pellet of simultaneous inoculation based co-culture (TB2C) Culture pellet of sequential inoculation based co-culture

Heat maps showed the differential expression of metabolites between the axenic and co-culture of both intra and extra cellular level (Fig. 5). It was generated using the normalized data of the axenic and co-culture. The results showed that the metabolites of *B. amyloliquefaciens* and *T. asperellum* were induced in the co-culture of TB1 and TB2, respectively. In the supernatant of TB1, cis-Aconitic acid, uric acid, cysteine and citric acid were increased compared to the B. In intracellular, mannitol, 5-aminovaleric acid, and arabinose were increased compared to the B. Glutamic acid, aspartic acid, phosphoric acid, 2,4-dihydroxybutanoic acid, 2-hydroxyglutaric acid, 2-ketoglutaric acid, galactose, tryptophan, pyroglutamic acid, maltose, uracil, glucose and glucaric acid were increased in TB2 compared to the T, whereas 48 different metabolites were upregulated in the TB2 compared to T at intracellular level.

Investigation on KEGG was carried out to compare changes between the metabolic pathway of axenic (T and B) and co-culture (TB1 and TB2) in both extracellular and intracellular level (Additional file 1: Fig. S3). 54 metabolic pathways showed major variations between TB2 and T. Among them, 12 were associated with amino acid metabolism, and others such as 4 carbohydrate metabolism, 2 nucleic acid metabolism, tropane, piperidine and pyridine alkaloid biosynthesis, C5-Branched dibasic acid metabolism, 2-Oxocarboxylic acid metabolism, carbon metabolism, pantothenate and CoA biosynthesis, inositol phosphate metabolism, streptomycin biosynthesis, taurine and hypotaurine metabolism, glycerophospholipid metabolism, sulfur metabolism, methane metabolism, biosynthesis of antibiotics, butanoate metabolism, carbon fixation pathways in prokaryotes, microbial metabolism in diverse environments, xylene degradation, atrazine degradation, phenylpropanoid biosynthesis, biosynthesis of secondary metabolites, glyoxylate and dicarboxylate metabolism, benzoate degradation, aminoacyl-tRNA biosynthesis, nicotinate and nicotinamide metabolism and galactose metabolism. Similarly, In TB1 55 metabolic pathway including 7 carbohydrate metabolism, 16 amino acid metabolism, tropane, piperidine and pyridine alkaloid biosynthesis, C5-Branched dibasic acid metabolism, pyrimidine metabolism, inositol phosphate metabolism, streptomycin biosynthesis, carbon metabolism, butanoate metabolism, glycerophospholipid metabolism, pantothenate and CoA biosynthesis, microbial metabolism in diverse environments, sulfur metabolism, xylene degradation, atrazine degradation, biosynthesis of antibiotics, carbon fixation pathways in prokaryotes, 2-oxocarboxylic acid metabolism, glyoxylate and dicarboxylate metabolism, biosynthesis of secondary metabolites, novobiocin biosynthesis, benzoate degradation, methane metabolism, purine metabolism, carbon fixation in photosynthetic organisms, taurine and hypotaurine metabolism, ascorbate and aldarate metabolism, monobactam biosynthesis, isoquinoline alkaloid biosynthesis, glycerolipid metabolism, biosynthesis of vancomycin group antibiotics, degradation of aromatic compounds, propanoate metabolism, aminoacyl-tRNA biosynthesis and nicotinate and nicotinamide metabolism were upregulated compared to the *Trichoderma* metabolic pathway. 21 and 64 pathways of TB1 and TB2 were upregulated when compared to the *Bacillus* axenic culture.

### Inhibition of *F. graminearum* growth by the axenic and co-culture

The effect of axenic and co-culture fermentation liquor on the growth inhibition of *F. graminearum* was defined by the growth inhibition assay. The growth of *F. graminearum* in the PDA medium supplemented with TB2 liquor was decreased to 80.1%, followed by TB1 (71.5%), T (48.6%) and B (45.3%) (Additional file 1: Fig. S4).

### Maize seed germination assay

**Fig. 6:**
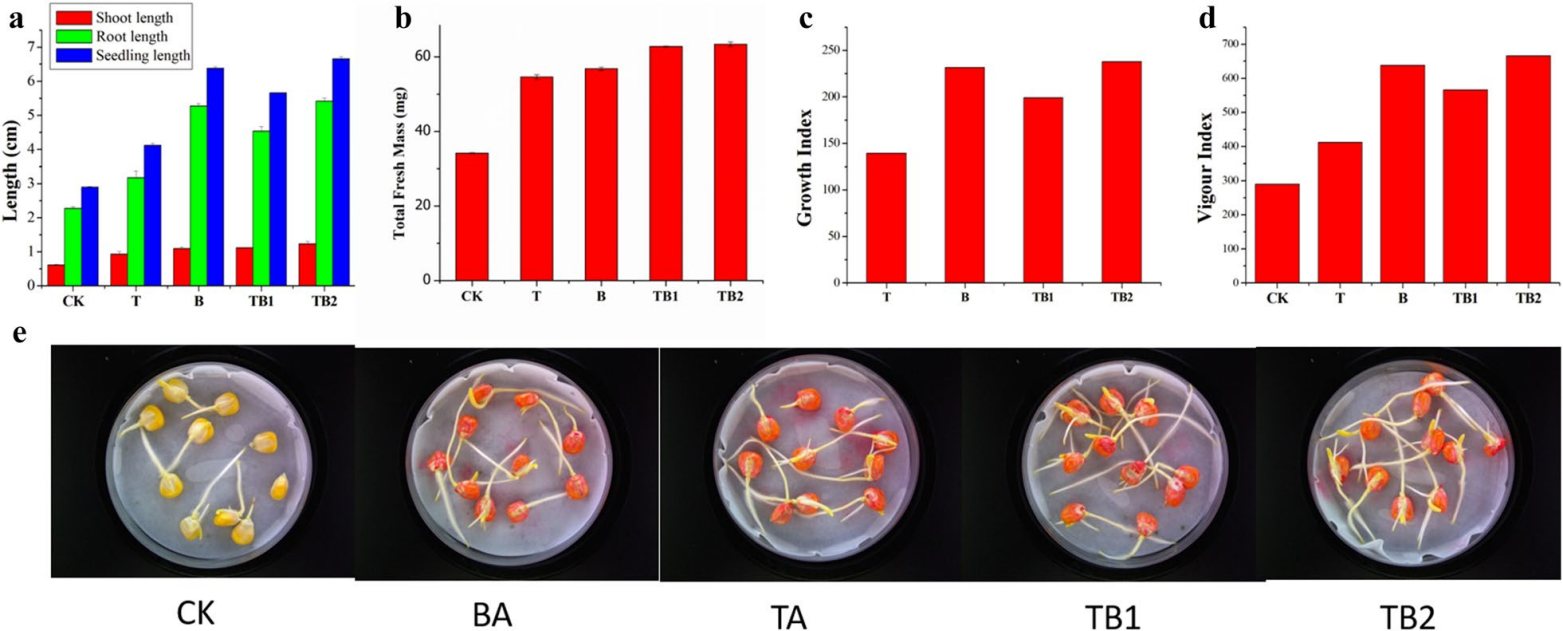
Effect of axenic (T and B) and co-culture (TB1 and TB2) of *T. asperellum* and *B. amyloliquefaciens* on maize seed germination. **a** Shoot, root and total seedling length of germinated seeds. **b** Total fresh weight of the germinated seeds. **c** Growth index of the germinated seeds. **d** Vigour index of the germinated seeds. **e** Maize seed germination differences between each treatment. (CK) control; (BA) axenic culture of *B. amyloliquefaciens* (TA) axenic culture of *T. asperellum* (TB1) simultaneous inoculation based co-culture (TB2) sequential inoculation based co-culture. Results are means of 10 replicates for each treatment; the value is the standard error of the mean. Different letters above the bars are significantly different (P < 0.05) based on the ANOVA

Our results highlight the significant role of co-culture on maize seed germination. The positive effects of the axenic and co-culture of *T. asperellum* and *B. amyloliquefaciens* on seed germination are shown in Fig. 6. The parameters such as root length, shoot length, total seedling length, total seedling fresh mass, growth index and vigor index were improved significantly compared to the control. The seedling length of the seeds treated with TB2 and TB1 were higher than the other treatments, whose root length, shoot length and total seedling length values ranged from 4.54 to 5.42 cm, 1.12 to 1.24 cm and 5.66 to 6.66 cm, respectively. The fresh mass of the seeds treated with TB1 and TB2 were superior then the axenic culture, presenting 62.8 and 63.4 mg, respectively (Fig. 6). The control showed the lowest weight. The vigor index of the seeds treated with TB1 (566) and TB2 (666) were higher than other treatments. In this sequence, the axenic cultures of T and B were superior to the negative control (290) with vigor index ranging from 412 to 638 (Fig. 6).

### Enhancement of biocontrol activity and Maize Plant growth by the co-culture of *T. asperellum* and *B. amyloliquefaciens*

Growth-promoting and bio-control effects of the axenic and co-culture on maize plants were observed based on the root length, shoot length, fresh and dry weight of root and shoot and disease reduction. As shown in Table 1, the maize plants treated with the axenic (T and B) and co-culture of *T. asperellum* and *B. amyloliquefaciens* (TB1 and TB2) either infested or un-infested with *F. graminearum* significantly improved the plant growth compared to control. Besides, co-culture (TB1 and TB2) significantly triggered the maize growth and development compared to treatment of axenic culture (T and B). The plants treated with both axenic and co-culture improved the height of maize plant under infected and un-infected soil. The co-culture increased the shoot and root length compared to axenic culture. Though, the sequential inoculation based co-culture showed the maximum shoot length either in infected (117.6 cm) or in un-infected soil (119.8 cm). The co-inoculation based co-culture showed the maximum root length in both infected (59.1 cm) and un-infected soil (57.8 cm). As shown in Table 1, the shoot and root biomass of the plants treated with axenic and co-culture increased significantly compared to the control. Likewise, co-culture (TB1 and TB2) significantly increased wet and dry weight of plant shoot and root compare to the axenic culture. However, the TB2 provided the highest wet and dry weight of the shoot either in infected (77.08 g and 6.02 g, respectively) or in un-infected soil (75.89 g and 6.95 g, respectively), while the treatment of TB1 provided the superior wet and dry weight of the root either in infected (25.60 g and 2.54 g, respectively) or in un-infected soil (24.04 g and 2.74 g, respectively). The treatment of maize infected with *F. graminearum* by the co-culture significantly increased the disease reduction compared to the axenic culture and infected control (FG). Consequently, T and B significantly reduced the infection of *F. graminearum* in maize and improved the plant growth.

**Table 1:** Effect of axenic (T and B) and co-culture (TB1 and TB2) of *T. asperellum* and *B. amyloliquefaciens* on the plant growth and biological control of *F. graminearum* under green-house conditions

Treatments	Shoot length (cm)	Root length (cm)	Wet weight (shoot) (g)	Wet weight (root) (g)	Dry weight (shoot) (g)	Dry weight (root) (g)	Disease reduction (%)
T1	93.43 ± 0.14^f^	52.1 ± 0.39^c^	59.77 ± 0.34^d^	22.272 ± 0.37^cd^	5.586 ± 0.24^bcd^	3.73 ± 0.36^a^	NA
T2	92.22 ± 0.17 ^g^	54.76 ± 0.44^b^	53.692 ± 0.34^e^	22.64 ± 0.26^cd^	5.28 ± 0.24^cde^	2.81 ± 0.18^a^	NA
T3	110.68 ± 0.17^c^	57.88 ± 0.35^a^	66.894 ± 0.38^c^	26.334 ± 0.30^a^	5.768 ± 0.23^bcd^	2.886 ± 0.21^a^	NA
T4	119.82 ± 0.38^a^	56.2 ± 0.32^b^	75.898 ± 0.44^a^	24.048 ± 0.38^abc^	6.956 ± 0.37^a^	2.74 ± 0.27^a^	NA
T5	98.3 ± 0.23^e^	48.42 ± 0.32^d^	56.438 ± 0.47^e^	23.664 ± 0.40^bcd^	6.26 ± 0.14^bcd^	2.364 ± 0.14^a^	70^b^
T6	89.54 ± 0.24^h^	55.72 ± 0.52^b^	52.12 ± 0.39^f^	21.602 ± 0.22^d^	4.89 ± 0.15^d^	2.386 ± 0.22^a^	70^b^
T7	107.86 ± 0.3^d^	59.12 ± 0.48^a^	59.646 ± 0.27^d^	25.602 ± 0.48^ab^	6.334 ± 0.41^abc^	2.544 ± 0.30^a^	90^a^
T8	117.66 ± 0.27^b^	55.48 ± 0.32^b^	72.084 ± 0.33^b^	26.318 ± 0.54^a^	6F.026 ± 0.35^ab^	3.48 ± 0.24^a^	90^a^
T9	43.48 ± 0.17^i^	36.7 ± 0.34^f^	12.462 ± 0.30 ^h^	4.724 ± 0.44^f^	1.354 ± 0.22^f^	0.898 ± 0.43^c^	40^c^
T10	76.22 ± 0.24^j^	41.18 ± 0.57^e^	47.348 ± 0.42^g^	13.268 ± 0.4^e^	3.8 ± 0.38^e^	1.692 ± 0.19^b^	NA

Results are means of 5 replicates for each treatment; the value is the standard error of the mean

NA represents not applicable

Different superscripts in the same column are significantly different (P < 0.05) based on the ANOVA. (T1)—T (*Trichoderma asperellum* GDFS1009); (T2)—B (*Bacillus amyloliquefaciens* 1841); (T3)—TB1 (co-inoculated *T. asperellum* GDFS1009 + *B. amyloliquefaciens* 1841); (T4)—TB2 (Sequential inoculated *T. asperellum* GDFS1009 + *B. amyloliquefaciens* 1841); (T5)—T + FG (Challenged with *Fusarium graminearum*); (T6)—*B *+ *FG*; (T7)—TB1 + FG; (T8)—TB2 + FG; (T9)—FG; (T10)—Control

Consistently, a large number of *Trichoderma* and *Bacillus* were developed under the root surface. The highest number of *Bacillus* spores was re-isolated from the rhizosphere soil of plants treated with B in un-infected soils. Followed by, TB1 and TB2 treatment on both infected and un-infected soil showed the presence of *Bacillus* in the rhizosphere. Similarly, the *Trichoderma* was found highest in the plants treated with T in un-infected soil (TA), followed by TB1, TB2, TB1 + FG and TB2 + FG. Whereas, the highest number of *Fusarium* colonization was observed in the control of infected soil (FG). The lowest number of *Fusarium* colonization was observed in TB1 + FG and TB2 + FG (Table 2). Whole together, the in vivo experiments revealed the root colonization of *Trichoderma* and *Bacillus* could induce the maize systemic disease resistance against the *F. graminearum*. The proportion of bio-control agent relative to pathogen in the rhizosphere of the plants treated with axenic and co-cultures under biotic stress was calculated. The results showed that the proportion of BC: P was higher in the plants treated with the TB2. While, TB1 was lesser than the B (Table 2).

**Table 2:** Re-isolation of *Trichoderma*, *Bacillus* and *Fusarium* from the soils treated with the axenic (T and B) and co-culture (TB1 and TB2) of *T. asperellum* and *B. amyloliquefaciens* under normal and biotic stress conditions

	T (CFU)	B (CFU)	TB1 (CFU)	TB2 (CFU)	T + FG (CFU)	B + FG (CFU)	TB1 + FG (CFU)	TB2 + FG (CFU)	FG (CFU)	Control (CFU)
*Bacillus* count	ND	13 × 10^8^ ± 0.31^a^	11 × 10^7^ ± 0.31^b^	6.6 × 10^6^ ± 0.48^c^	ND	2 × 10^8^0.31^d^	8 × 10^6^ ± 0.31^c^	3.3 × 10^6^ ± 0.31^d^	ND	ND
Other bacterial count	4.5 × 10^7^ ± 0.15 ^cd^	ND	ND	24.3 × 10^6^ ± 0.48^a^	13 × 10^6^ ± 0.31^b^	ND	ND	5.4 × 10^6^ ± 0.49^c^	8 × 10^8^ ± 0.31^d^	3 × 10^8^ ± 0.31^d^
*Trichoderma* count	4.1 × 10^5^ ± 0.3^b^	ND	1.63 × 10^3^ ± 0.09^c^	3 × 10^5^ ± 0.31^b^	6 × 10^6^ ± 0.31^a^	ND	3.4 × 10^4^ ± 0.23^b^	3.2 × 10^5^ ± 0.22^b^	ND	ND
Other fungal count	1.3 × 10^2^ ± 0.18^e^	3.1 × 10^2^ ± 0.23^d^	8.6 × 10^3^ ± 0.23^a^	8.6 × 10^2^ ± 0.18^a^	5.9 × 10^2^ ± 0.11^b^	3.2 × 10^2^ ± 0.14^d^	5.0 × 10^2^ ± 0.09^c^	6.2 × 10^2^ ± 0.04^b^	8.2 × 10^5^ ± 0.1^c^	4.2 × 10^4^ ± 0.09^c^
*Fusarium* count	ND	ND	ND	ND	4 × 10^2^ ± 0.31^a^	2.9 × 10^2^ ± 0.22^b^	0.3 × 10^1^ ± 0.031^c^	0.15 × 10^1^ ± 0.02^bc^	8.6 × 10^6^ ± 0.48^d^	ND
BC: P proportion	NA	NA	NA	NA	15,000 ± 0.45^d^	689,655.1724 ± 0.37^b^	267,800 ± 0.24^c^	2,413,333.333 ± 0.11^a^	NA	NA

Results are means of 3 replicates for each treatment; the value is the standard error of the mean. Values below the detectable levels are indicated in the graphs as ND (not detected); NA (not applicable)

*ND* not detectable, *NA* not applicable

Different superscripts in the same column are significantly different (P < 0.05) based on the ANOVA

**Fig. 7:**
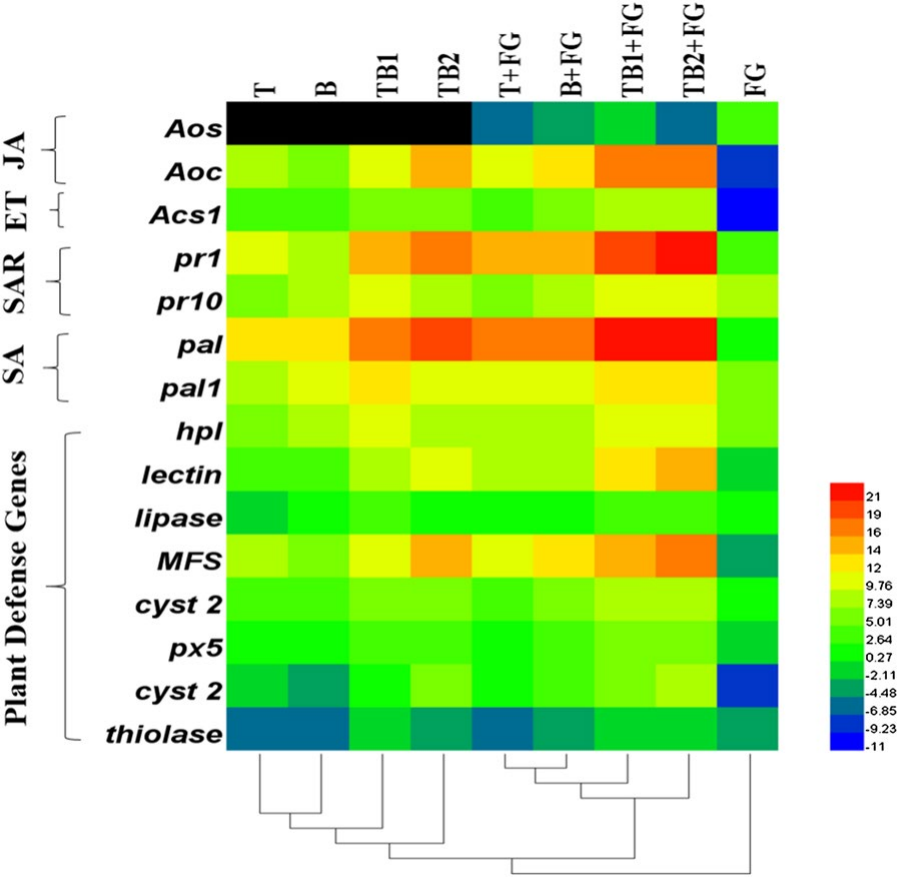
Differential expression profiles of defense-related genes in maize root tissues treated with axenic (T and B) and co-culture (TB1 and TB2) of *T. asperellum* and *B. amyloliquefaciens* under normal and biotic stress (*F. graminearum* infected soil (FG)) conditions. Relative transcript abundance was determined using RT-qPCR. Data are expressed as mean ± standard error of three replicates. *JA* jasmonic acid related response; *ET* ethylene related response; *SAR* systematic acquired resistance related response; *SA* salicylic acid related response

The expression of maize defense genes on the roots were investigated to know the effect of axenic and co-culture on regulation of maize disease resistance against pathogen (Fig. 7). The expression of allene oxide synthase (*AOS*) gene belongs to the jasmonic acid pathway was significantly changed among the different treatments. Expression of *AOS* gene was higher in the plants infested with *F. graminearum* (T7) and it was steadily decreased in the infested plants treated with co-culture (TB2 and TB1) and axenic culture (T and B). The Cq for the *AOS* gene was not detected in the non-infested plants. The expression of genes such as *AOC* and *ACS* involved in the ethylene pathway was studied. The *AOC* and *ACS* genes were up-regulated in TB1 + FG and TB2 + FG, followed by TB1 and TB2. Similarly, the axenic cultures (T and B) also induced the expression of *AOC* and *ACS* genes in both infested and un-infested plants, while the expression of these genes were downregulated in the FG. The expression of *PR1* and *PR10* involved in the systemic acquired resistance pathway (SAR) was studied. Among the different treatments, *PR1* and *PR10* were up-regulated in the T8 (FG + TB2) T7 (FG + TB1), T3 (TB1) and T4 (TB2) followed by T5 (FG + T), T6 (FG + B), T1 (T) and T2 (B). Similarly, the *PAL* and *PAL1* genes involved in salicylic acid pathway was also induced in the T8 (FG + TB2) T7 (FG + TB1), T3 (TB1) and T4 (TB2), followed by T5 (FG + T), T6 (FG + B), T1 (T) and T2 (B). The gene expressions of *HPL*, lectin, lipase, *MFS*, *Cyst2*, *PX5*, *Cyst* and thiolase were also induced in T8 and T7 (Fig. 7), while these genes were downregulated in T9.

## Discussion

*Trichoderma* and *Bacillus* have been proved as an effective candidate for controlling the plant diseases and growth promotion. Several research has defined the application of *Bacillus* either independently or in blend with additional microbes like *Trichoderma* to control the plant pathogens [22–24]. *Trichoderma* and *Bacillus* could propagate in a broad range of pH and soil. They produce hydrolytic enzymes, secondary metabolites and plant growth promoting components to enhance the plant growth and defense potential. Microbial genome mining revealed that secondary metabolite biosynthetic gene cluster remains silent in most of the microorganisms. This statement stimulates the idea to activate these silent gene using different methods such as modification of culturing technology, ribosome engineering, exchange and regulation of promoters, and synthetic biology-based pathway alteration [25, 26]. Modification in the culture conditions has been considered as the modest approach [25]. Hence, In the present investigation the co-cultivation of *Trichoderma* and *Bacillus* were carried out using sequential and co-inoculation based method to induce the production of secondary metabolites and plant growth promoting compounds.

It is worth to note growth rate of *T. asperellum* and *B. amyloliquefaciens* in co-inoculation and sequential inoculation treatments. The results showed a negative effect on the growth of *T. asperellum* in the co-inoculation. Faster growth of *Bacillus* over *Trichoderma* resulted in declined growth rate of *Trichoderma* in the co-inoculation method. The inoculation of *Bacillus* in the 48 h grown *Trichoderma* pre-culture promoted the growth of both microbes.

Interactions of *Trichoderma* with other microorganisms could alter the pattern of gene expression. Karuppiah et al. [21] reported that several genes have been upregulated during co-cultivation. Hence in the present investigation, the gene expression of *Trichoderma* spp. under the different co-cultivation conditions have been studied. The genes such as *BLR1* and *BLR2*, *ENV1*, *Vel1*, *TMKa* and *GPR1* involved in the sporulation’s were upregulated in the sequential based co-culture, while these genes were downregulated in the co-inoculation based co-culture. These results are in agreement with the spore count of axenic, sequential and co-inoculation based co-culture. The regulation between the expression of blue-light-regulated genes (*BLR1* and *BLR2*), ENVOY (*ENV1*), velvet (*Vel1*) mitogen-activated protein kinase (*TMKa*) and G protein receptor 1 (*GPR1*) are in agreement with the Karuppiah et al. [21] and Carreras-Villasenor et al. [27].

Secondary metabolites produced by microbes could be affected under the laboratory culture condition [28]. Depending on the communication between the microbes in co-culture, several genes could activate the production secondary metabolites. In the present investigation, non-ribosomal peptide synthetase (*NP1* and *NP2*), Putative ferrichrome synthetase (*NP3*), Cytochrome P450 (*Tri* 13) 1, *OMT* and Polyketide synthetase (*PK1* and *PK2*) genes of *Trichoderma* involved in the secondary metabolite synthesis were up-regulated in sequential based co-culture, while these genes were downregulated in the co-inoculation based co-culture. *B. amyloliquefaciens* synthesize the polyketide compounds and involved in biocontrol activity [29]. In the present investigation *Loa P*, *DFN a*, *DFN g*, *DFN m* (difficidin operon) *MLN* a, *MLN* d, *MLN* i (macrolactin operon) were induced in the co-inoculation based co-culture. This results revealed the co-cultivation of *T. asperellum* and *B. amyloliquefaciens* was an appropriate technique to induce the secondary metabolites.

In-addition, the secretion of antioxidnats by *Trichoderma* in response to the *Bacillus* have been accessed using the quantification of catalase and *NADPH* oxidase gene. It has been established that *NOX* proteins are an important enzyme for sporulations [30]. The ROS scavenging systems such as ascorbate peroxidases, glutathione, superoxide dismutases, and catalases of *Trichoderma* are associated with NADPH oxidases to preserve the ROS homeostasis [30]. In the present study sequential inoculation based co-culture induced the expression of catalase and NADPH oxidase gene compared to the axenic culture. Further, the *NOX* also involved in the activation of proteases, together with aspartic, subtilisin serine, and trypsin-like proteases in *T. harzianum* T34 [30]. Similarly, we observed the induction of genes encoding aspartyl protease, trypsin-like protease, chitinase, β-1, three-glucanase, β-1,6-glucanase, β-1,4-glucanase and *N*-acetyl-glucosaminidases in the sequential based co-culture.

Co-cultivation influences the metabolic profile of the another microbe. The present investigation demonstrated that co-culture not only generate new metabolites, it also generates the metabolites of both microbes with different quantity. In TB1, the *Bacillus* reduced the quantity of some *Trichoderma* metabolites. Similarly, in TB2, *Trichoderma* downregulated the production of some *Bacillus* metabolites. Pyroglutamic acid, a non-protein amino-acid was induced in TB2. The induced production of glutamic acid and glutamine in the TB2 might be responsible for the synthesis of pyroglutamic acid [31].

Amino acids have several uses in pharmaceutical and agriculture application [32–34]. Interestingly, in the present investigation, amino acids covered up to 26–39% of the total metabolites. 5 amino acids including aspartic acid, homoserine, alanine, isoleucine and methionine were significantly increased in B. Similarly, asparagine and serine were significantly increased in T. The amino acids such as histidine, lysine, phenyl alanine, proline, tryptophan and tyrosine were significantly increased in TB1. Glutamine and aspartic acid was found to be significantly high in TB2. The amino-acids produced by *Bacillus* were detected in TB2, but the level of production was significantly reduced compared to the TB1 and B.

Atilio and Causin et al. [35] reviewed that amino acid is an essential component for the plant growth and metabolism. They also help the plants to grow under the biotic and abiotic stresses [36–38]. Under stress conditions, amino acids were catabolized through tricarboxylic acid (TCA) cycle and generate energy requisite for the growth and development [39]. In TB2, the metabolites of TCA cycle, glycolysis and sugars such as glucose and galactose were increased be due to the catabolism of amino acids generated by the *Bacillus* in co-culture (TB2) and this might be the reason for the lowest concentration of amino acids in TB2. Further, the maize growth under greenhouse conditions showed that these amino acids have been utilized by the plants to grow higher than that of axenic culture under normal and biotic stress conditions.

Some bacteria synthesize vitamin B to stimulate the plant growth [40–42]. In the present study, TB1 produced higher amount of pantothenic acid, and thiamine compared to the B, while in TB2 it was reduced. Thiamine is an important cofactor for the biosynthesis of indole acetic acid in plants and also required for several essential metabolisms [43, 44]. Foliar application of thiamine increased the vegetative growth and chemical constituents of plants [45]. The nicotinic acid improved the root length of the plant [46]. Similarly, in the present investigation the root length of the maize plants treated with TB1 was increased under both normal and biotic stress conditions.

Pipecolic acid detected in TB1 was abundant compared to B. It was found to be a key element to establish the systemic acquired resistance pathway in plants [47]. The 4-aminobutyric acid was induced in the both TB1 and TB2. 4-aminobutyric acid is a non-protein amino acid, which was regularly accumulated in plants during ecological stress and there by induce ethylene production [48, 49]. It also helps the plant to grow under environmental stresses [49, 50]. It also reduced the growth rate of larvae and stimulated the plant growth [51]. Similarly, the trans ferulic acid was found to be induced in TB1 compared to other groups. The trans ferulic acid had a potential to induce plant resistance [52]. Moussa et al. [53] observed that the plants treated with ethanolamine under salt stress were highly adapted through the highest ROS scavenging photosynthetic activity. In present study the ethanolamine was detected in both TB1 and TB2. The presence of these metabolites are correlated well with the induction of maize plant defense genes related to SAR and salicylic acid pathway. The organic acids such as lactic acid, malic acid and citric acid were found to be highest in TB2. These organic acids have the antimicrobial activity against *Listeria monocytogenes*, *Escherichia coli*, and *Salmonella gaminara* [54]. In addition, these organic acids solubilize the soil inorganic phosphates to induce the plant growth [55, 56]. These organic acids present in the co-cultures might be involved in the reduction of *F. graminearum* in the soils treated with co-cultures (Table 2). Further, the highest production of organic acids in TB2 increased the proportion of BC: P of plants treated with TB2 under biotic stress conditions. The results revealed that the TB2 was more active against the soil pathogens compared to the TB1.

## Conclusions

The inoculation sequence proved the competitive interaction between *T. asperellum* and *B. amyloliquefaciens*, which also influenced growth rate, differential expression of vital genes and metabolites during the fermentation. The competitive interaction between the *T. asperellum* and *B. amyloliquefaciens* in co-inoculation method downregulated the expression of *Tricoderma* vital genes and metabolites, while in sequential inoculation the didicidin and macrolactin gene expression and metabolites of *Bacillus* were downregulated. On the other hand, it has been predicted that aminoacids produced by *Bacillus* might be utilized by *Trichoderma* for its energy through TCA cycle in TB2. From this study it has been observed that metabolic and genetic expression of *T. asperellum* and *B. amyloliquefaciens* could be induced by the simultaneous and sequential inoculation based co-cultivation method, respectively. Even though the competitive interaction occurred in the simultaneous and sequential inoculation, it favored the maize growth and bio-control activity in different ways. Taken together, the results suggested that the metabolites generated by the co-culture helps the maize plant to grow under normal and biotic stress conditions by (1) providing the nutrients such as carbon and nitrogen for plant growth, (2) improve the plant resistance by the activation of the defense related genes and (3) inhibit the growth of soil pathogens through the secretion of antimicrobial metabolites. Thus in future, the co-cultivation based microbial consortia could be used as a new technology to increase the plant growth and development.

## Methods

### Microbes and culture conditions

The *T. asperellum* GDFS1009 and *B. amyloliquefaciens* 1841 were procured from China General Microbiological Culture Collection Center and Laboratory of Microbial Fermentation, Sichuan University, China, respectively. The fungus was recovered and grown on Potato Dextrose (PD) medium at 28 °C. The *B. amyloliquefaciens* 1841 was recovered and grown on nutrient medium at 37 °C. The *T. asperellum* GDFS1009 and *B. amyloliquefaciens* 1841 grown in PD and nutrient medium were used as inoculum for all investigations. *Fusarium graminearum* (maize stacks and root rot pathogen) was used as the test pathogen for biocontrol activity.

### Co-cultivation of *T. asperellum* (GDFS1009) and *B. amyloliquefaciens* 1841

Sequential inoculation: 1% of *T. asperellum* GDFS1009 was pre-cultured at 28 °C for 48 h in 100 mL of YMC broth [21]. Consequently, 0.1% of the *B. amyloliquefaciens* 1841 (1.0 OD at 600 nm) was inoculated into the pre-culture medium and continued the co-cultivation for 2 days at 28 °C. Co-inoculations: 1% of *T. asperellum* GDFS1009 and 0.1% of *B. amyloliquefaciens* 1841 were co-inoculated into 100 mL YMC broth and incubated in shaker for 4 days at 28 °C. After incubation, the broth cultures were serially diluted and plated in the PDA containing streptomycin and chloramphenicol to estimate the growth of fungus. The bacterial growth was estimated using the nutrient agar containing nystatin and cycloheximide [21].

### Enzyme activity

The axenic (T and B), sequential (TB2) and co-inoculation (TB1) of co-cultures were centrifuged and the supernatants were used as the crude protein extract. The enzyme activity such as chitinase, neutral protease, β-1,3-glucanase and cellulase were estimated using Enzyme Activity Determination Kits (Shanghai Cablebridge Biotechnology Co., Ltd.), according to the manufacturer’s protocol [21].

### Gene expression

The expression of genes associated with sporulation (*VEL* 1, *TMK*, *GPR* 1, *BLR* 1, *BLR* 2, and *ENV1*), mycoparasitism related enzymes (*NAG* 1, *NAG* 2, *PAP* A, *PAP* B, *ECH*, *AF*, *ACC*, *BGN 13*, *BGN* 16, and *EG1*) secondary metabolism (genes encoding three *NRPSs*, two *PKSs*, O-*methyltransferase* B, and cytochrome P450) and antioxidants (*NOX* and *CAT*) of *Trichoderma* [21], macrolactin and difficidin genes [29] of *B. amyloliquefaciens* were studied in the axenic and co-culture of the *T. asperellum* and *B. amyloliquefaciens* grown in the YMC medium at 4th day.

RNA was extracted as defined previously [21]. For expression analysis, RNA was extracted using TRIeasy total RNA extraction reagent (YEASEN). cDNA was generated according to the procedure of Prime Script RT reagent kit with DNA eraser (Takara). The real time PCR and relative quantification was performed as described by Karuppiah et al. [21]. The primers used for gene quantification was given in Additional file 1: Table S1.

### Preparation of samples for LC–MS/MS

50 mL of each culture supernatant BCS, TCS, TB1CS and TB2CS, and its pellet containing microbes BC, TC, TB1C and TB2C alongside 50 mL of YMC medium as controls, were snap-frozen with fluid nitrogen and transported on dry ice to the Suzhou BioNovoGene Metabolomics Platform. All of them were defrosted at 4 °C and blended consistently. 200 µL of each sample was taken in a 1.5 mL microcentrifuge tube, to which 800 µL of methanol was added and vortexed for 60 s and centrifuged at 12,000 RPM at 4 °C for 10 min. The supernatants were exchanged to another 1.5 mL micro-centrifuge tube, dried and mixed in 300 µL of 80% methanol. Next, the supernatants were separated through a 0.22 µm film to acquire the examples for analysis. The samples were analyzed through Waters ACQUITY UPLC with ACQUITY UPLC^®^ BEH C18 1.7 µm (2.1 × 100 mm) chromatography column and mass spectrometry using Thermo LTQ Orbitrap XL instrument with electrospray ionization (ESI) and cation–anion ionization mode as described by Wu et al. [20].

### Analysis of metabolome data

The information on the data preprocessing were followed as described by Wu et al. [20], The raw data acquired was changed into the mzXML format by utilizing a Proteowizard program (v3.0.8789). The XCMS tool of R (v3.3.2) was used for peak detection, filtration and alignment. The significant parameters included bw = 5, ppm = 15, peak width = c (10, 120), mzwid = 0.015, mzdiff = 0.01 and strategy = ”centWave”. The data matrix containing the data of mass to charge proportion (m/z), retention time and peak area were obtained. The positive and negative particle models were assembled for 3680 and 3566 precursor molecules separately. The information was gathered and the accompanying investigations were carried out. The batch normalization of peak area was directed. Few multivariate statistical investigations such as principal component analysis (PCA) and Orthogonal Projections to Latent Structures Discriminant Analysis (OPLS-DA) were directed to uncover the distinctions in the metabolic creations between various groups. The metabolites with precise changes were analyzed in KEGG. The correlation between the metabolites were dissected by figuring the Pearson or Spearman rank correlation coefficient between any two metabolites. At the point when the straight connection between two metabolites expanded, the relationship coefficient drifted toward 1 or − 1 i.e., 1 represents a positive correlation and − 1 represents negative correlation. The statistical check was analyzed using cor.test in R package. Also, the false positive p-value was analyzed. FDR p-value ≤ 0.05 was utilized as the significant correlation [20].

### Determination of pathogen inhibition

The effect of axenic and co-culture on the inhibition of pathogen growth was determined according to the method of karuppiah et al. [21]. The PDA agar plates were prepared by mixing the filtered fermented broth of T, B, TB1 and TB2 in the ratio of 1:5. The *Fusarium graminearum* was loaded into the center of plates to access its growth inhibition by the axenic and co-cultures.

### Seed germination

Maize seeds were disinfected with 70% ethanol and 0.2% sodium hypochlorite for 2 min, followed by it was washed with sterile distilled water and air dried. 50 grams of maize seeds were treated with 2 mL of axenic and co-culture suspension (5 × 10^8^/mL) complemented with 0.15% of seed coating agent (Jilin Bada Pesticide Co. Ltd.). After the treatment, the seeds were distributed on the petri dish containing Whatman filter paper and germinated in the dark at 25 °C. The treatment with sterile distilled water was used as the control. The germination parameters such as root length, shoot length, total seedling length, total seedling fresh mass and vigor index were documented after 7 days.

### Plant growth and defense assay

Maize Seeds were planted into 30 cm diameter pots containing horticulture soil with 5 seeds per pot in 5 replications. Seedlings were thinned to two plants per pot after 1 week. The greenhouse experiment was carried out at day, night conditions; 28 °C for 12 h under lights, and at 24 °C in the dark for 12 h. The experiment was organized in a randomized complete blocks plan. After 2 weeks the plants were treated as follows: (T1)—T (*Trichoderma asperellum* GDFS1009); (T2)—B (*Bacillus* *amyloliquefaciens* 1841); (T3)—TB1 (co-inoculated *T. asperellum* GDFS1009 + *B.* *amyloliquefaciens* 1841); (T4)—TB2 (Sequential inoculated *T. asperellum* GDFS1009 + *B.* *amyloliquefaciens* 1841); (T5)—T + FG (Challenged with *Fusarium graminearum*); (T6)—*B *+ *FG*; (T7)—TB1 + FG; (T8)—TB2 + FG; (T9)—FG; (T10)—Control. The *T. asperellum* and *F. graminearum* inoculum were set at 1 × 10^6^ spores/mL while *B. amyloliquefaciens* inoculum was set at 1 × 10^8^ spores/mL. The axenic, sequential and co-inoculation of co-cultures were inoculated 5 days before and after inoculation with *F. graminearum*. The soil was moistened regularly. The plants were carefully uprooted and length of the root and shoot, wet and dry weight of the shoot and root were estimated.

The evaluation of disease was based on a scale of leaf spot disease from grade zero to grade five. Grade zero: no disease spot; Grade 1: no more than 10%; Grade 2: 11–30%; Grade 3: 31–50%; Grade 4: 51–70%; and Grade 5: more than 70% and the disease index was calculated according to the formula: Disease index = Σ (number of plants in each disease stage × levels value)/(total number of plants × the highest levels × 100). The disease reduction was computed with the following formula: Disease reduction = disease index of CK − disease index after treatment [57].

Total RNA was extracted from the plant root using the TRIeasy total RNA extraction reagent (YEASEN) and cDNA was generated according to the procedure of Prime Script RT reagent kit with DNA eraser (Takara). The expression of genes related to the jasmonic acid, ethylene (ET), systemic acquired resistance (SAR), salicylic acid (SA), plant innate immunity (PII) and other defense related genes were analyzed according to the method of Saravanakumar et al. [58]. The primers used for gene quantification is given in Additional file 1: Table S1. Relative gene expression was estimated by 2^ΔΔCT^ method using actin as a reference gene.

The inoculated *Bacillus*, *Trichoderma* and *Fusarium* were re-isolated from the rhizosphere soil of the treated plants. The rhizospheric soil of all treated plants were collected carefully after uprooting the plants. The rhizospheric soil was serially diluted and plated in the potato dextrose agar and nutrient agar to enumerate the fungi and bacteria, respectively. The percentage proportion of biocontrol agents (*Bacillus* and *Trichoderma*) relative to a pathogen (*Fusarium*) was calculated as follows:

Proportion of biocontrol agents: pathogen (BC: P) = Total CFU of bio-control agents/CFU of pathogen.

### Statistical analysis

All experiments were studied based on different replication and were repeated at least three times, with reproducible results. The graphs were constructed by means of Microsoft office excel and origin 6.0 with standard error bars. Results shown were average of replicates along with the standard error of mean values. For multiple comparisons, two-way ANOVA with post hoc LSD and Duncan were carried out using the SPSS 2.0. Student’s t-test was conducted to examine the differences between the gene expression using the SPSS 2.0. P < 0.05 was considered as significant. The heat map of defense gene expressions was made by means of HemI: A Toolkit for Illustrating Heat maps [59].

## Supplementary information

**Additional file 1.** Additional figures and table.

## Data Availability

All data generated during this study are included in this published article.

## Abbreviations

T: *Trichoderma asperellum*
B: *Bacillus amyloliquefaciens*
TB1: simultaneous inoculation based *T. asperellum* and *B. amyloliquefaciens* co-culture
TB2: sequential inoculation based *T. asperellum* and *B. amyloliquefaciens* co-culture
BCS: *B. amyloliquefaciens* culture supernatant
TCS: *T. asperellum* culture supernatant
TB1CS: *TB1* culture supernatant
TB2CS: *TB2* culture supernatant
BC: *B. amyloliquefaciens* culture pellet
TC: *T. asperellum* culture pellet
TB1C: *TB1* culture pellet
TB2C: *TB2* culture pellet
PR: pathogenesis related proteins
BCA: biocontrol agents
PD: potato dextrose medium
YMC: yeast extract, molasses and corn gluten meal medium
*Vel 1*: velvet
*TMKa*: mitogen-activated protein kinase
*GPR1*: G protein receptor 1
*BLR1*: blue-light-regulated genes I
*BLR2*: blue-light-regulated genes II
*NP1*: non-ribosomal peptide synthetase I
*NP2*: non-ribosomal peptide synthetase II
*NP3*: putative ferrichrome synthetase
*Tri* 13: cytochrome P450
*OMT*: 1,*O*-methyl transferase
*PK1*: polyketide synthetase I
*PK2*: polyketide synthetase II
*ENV1*: ENVOY
*Ech*: chitinase
*BGN13*: β-1,3-glucanase
*BGN16*: β-1,6-glucanase
*Egl*: β-1,4-glucanase
*NAG1*: *N*-acetyl-glucosaminidases 1
*NAG2*: *N*-acetyl-glucosaminidases 2
*PAP* A: aspartyl protease
*TLP* 1: trypsin-like protease
*ACC*: 1-aminocyclopropane-1-carboxylate deaminase
*NOX*: NADPH oxidases
*CAT*: catalase
*Loa P*: regulatory gene of difficidin and macrolactin operon
*DFN a*: difficidin A
*DFN g*: difficidin G
*DFN m*: difficidin M
*MLN a*: macrolactin A
*MLN d*: macrolactin D
*MLN i*: macrolactin i
*FG*: *Fusarium graminearum*
ET: ethylene
SAR: systemic acquired resistance
SA: salicylic acid
PII: plant innate immunity
P: pathogen
*AOS*: allene oxide synthase
*AOC*: allene oxide cyclase
*ACS*: 1-aminocyclopropane-1-carboxylic acid synthase
*PR1*: pathogenesis-related protein 1
*PR10*: pathogenesis-related protein 10
*PAL*: phenylalanine ammonia-lyase
*PAL1*: phenylalanine ammonia-lyase 1
*HPL*: hydroperoxide lyase
*MFS*: multiflux efflux synthase
*Cyst2*: cystatin ii proteinase inhibitor
*PX5*: peroxidase
*Cyst*: cystatin proteinase inhibitor
