# Supplementary material for: Simultaneous and sequential based co-fermentations of Trichoderma asperellum GDFS1009 and Bacillus amyloliquefaciens 1841: a strategy to enhance the gene expression and metabolites to improve the bio-control and plant growth promoting activity
Source: Microb Cell Fact. 2019 Oct 29;18:185. doi: 10.1186/s12934-019-1233-7 (PMC6819339; doi:10.1186/s12934-019-1233-7)
Supplement: Supplementary file 1 — Additional file 1. Additional figures and table. [file 12934_2019_1233_MOESM1_ESM.docx]

Figure. S1. Principal component analysis of the metabolic differences between the axenic (T and B) and co-culture (TB1 and TB2) of and *T. asperellum* and B. *amyloliquefaciens* based on LC–MS. **A** Extracellular based Metabolic Differences **B** Intracellular based Metabolic Differences. (TCS) Culture supernatant of *T. asperellum* axenic culture (BCS) Culture supernatant of *B. amyloliquefaciens* axenic culture (TB1CS) Culture supernatant of simultaneous inoculation based co-culture (TB2CS) Culture supernatant of sequential inoculation based co-culture (TC) Culture pellet of *T. asperellum* axenic culture (BC) Culture pellet of *B. amyloliquefaciens* axenic culture (TB1C) Culture pellet of simultaneous inoculation based co-culture (TB2C) Culture pellet of sequential inoculation based co-culture.


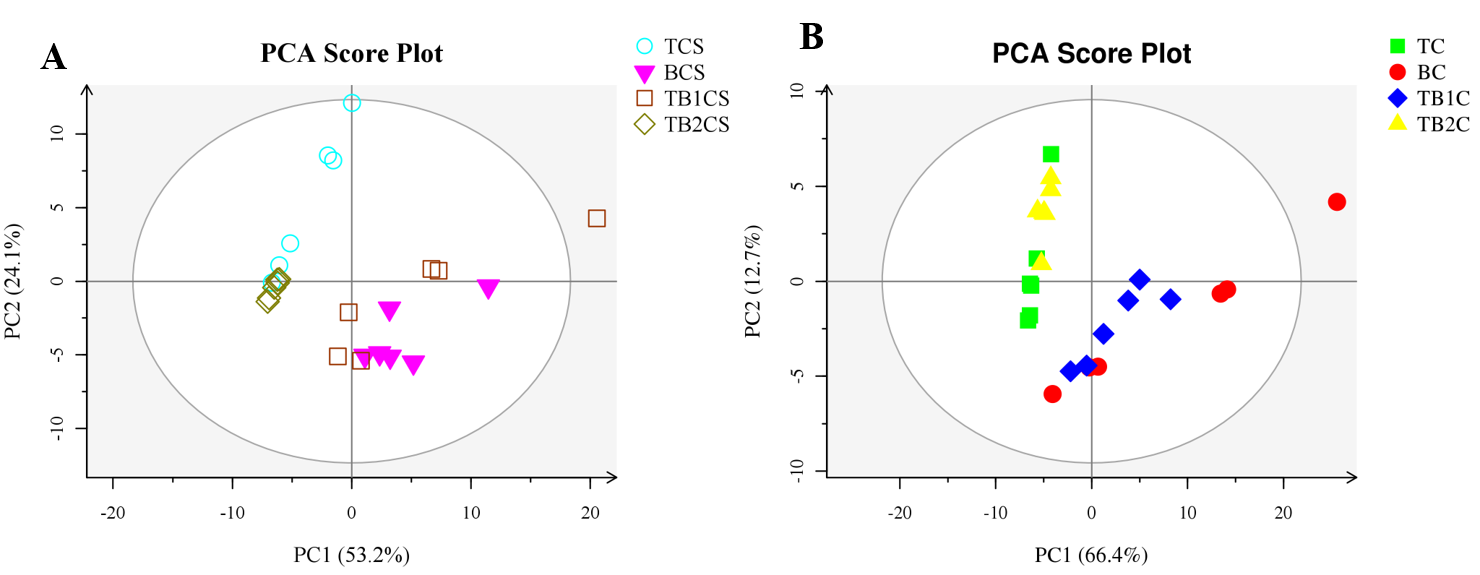


Figure S2. Metabolic differences between the axenic (T and B) and co-culture (TB1 and TB2) of and *T. asperellum* and B. *amyloliquefaciens* based on LC–MS. **A** Extracellular based Metabolic Differences **B** Intracellular based Metabolic Differences. (TCS) Culture supernatant of *T. asperellum* axenic culture (BCS) Culture supernatant of *B. amyloliquefaciens* axenic culture (TB1CS) Culture supernatant of simultaneous inoculation based co-culture (TB2CS) Culture supernatant of sequential inoculation based co-culture (TC) Culture pellet of *T. asperellum* axenic culture (BC) Culture pellet of *B. amyloliquefaciens* axenic culture (TB1C) Culture pellet of simultaneous inoculation based co-culture (TB2C) Culture pellet of sequential inoculation based co-culture.


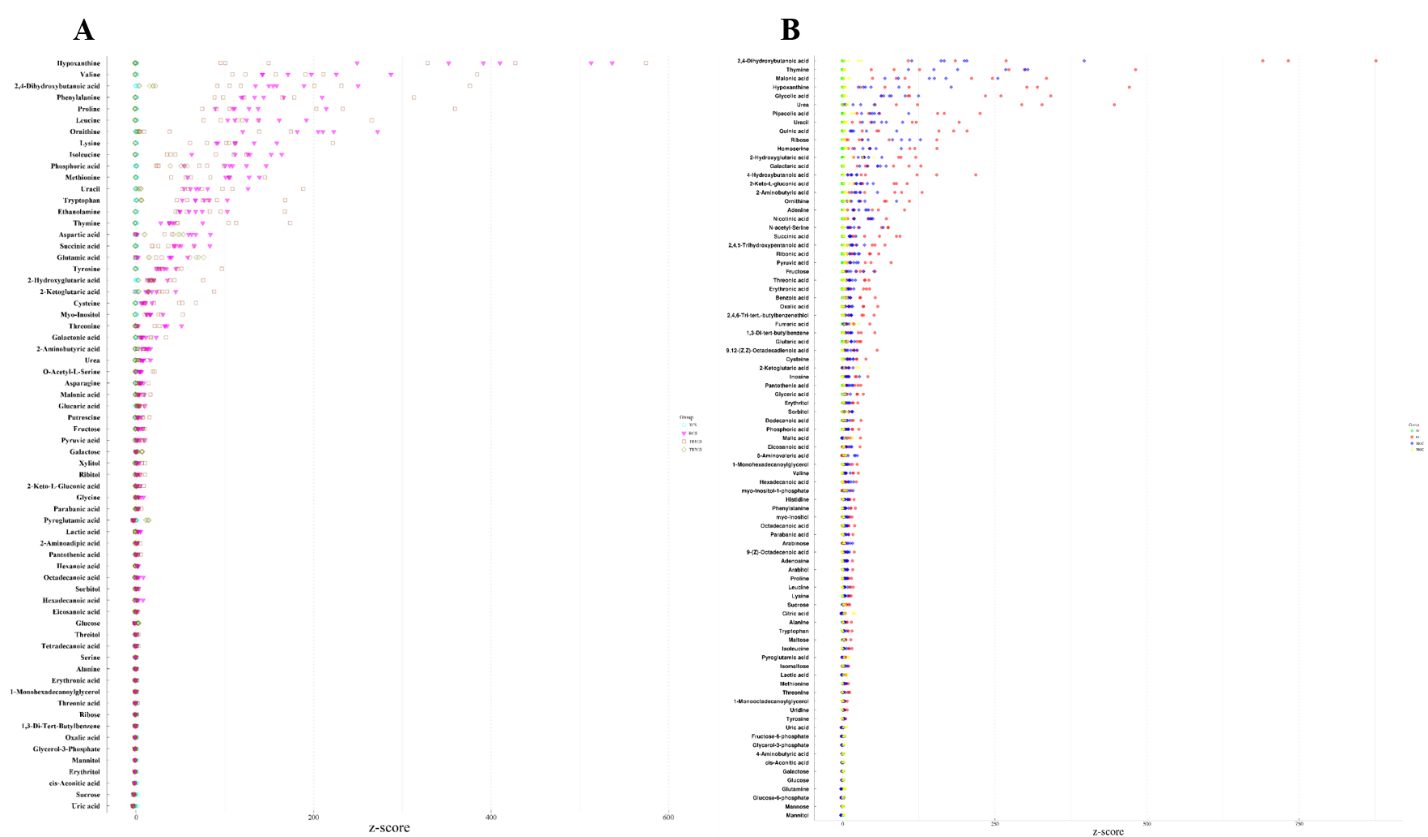


Figure. S3. KEGG pathway analysis on the metabolic differences between the axenic (T and B) and co-culture (TB1 and TB2) of and *T. asperellum* and B. *amyloliquefaciens* based on LC–MS. **A** Extracellular based Metabolic Differences **B** Intracellular based Metabolic Differences. (TCS) Culture supernatant of *T. asperellum* axenic culture (BCS) Culture supernatant of *B. amyloliquefaciens* axenic culture (TB1CS) Culture supernatant of simultaneous inoculation based co-culture (TB2CS) Culture supernatant of sequential inoculation based co-culture (TC) Culture pellet of *T. asperellum* axenic culture (BC) Culture pellet of *B. amyloliquefaciens* axenic culture (TB1C) Culture pellet of simultaneous inoculation based co-culture (TB2C) Culture pellet of sequential inoculation based co-culture.


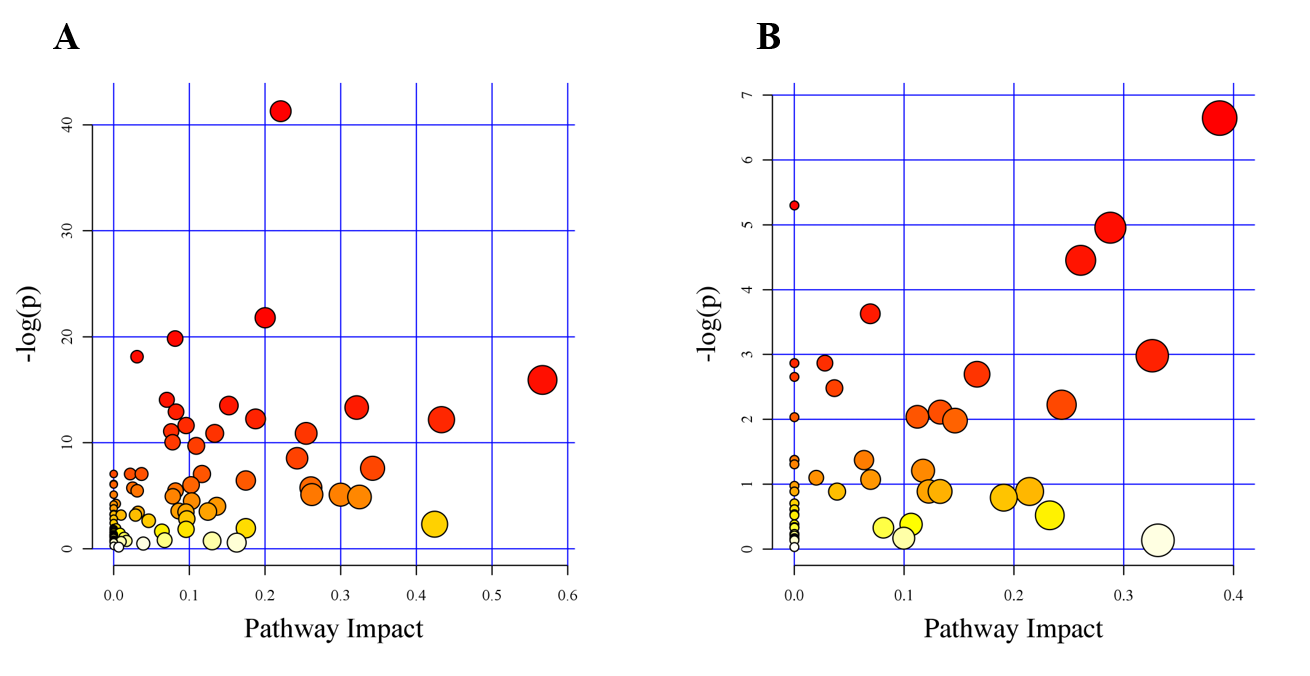


Figure S4. Growth inhibition of *F. graminearum* by the axenic (T and B) and co-culture (TB1 and TB2) of and *T. asperellum* and B. *amyloliquefaciens*. Results are means of five replicates for each treatment; the value in parentheses is the standard error of the mean. ∗represent significant differences between the axenic and co-culture (P < 0.05).


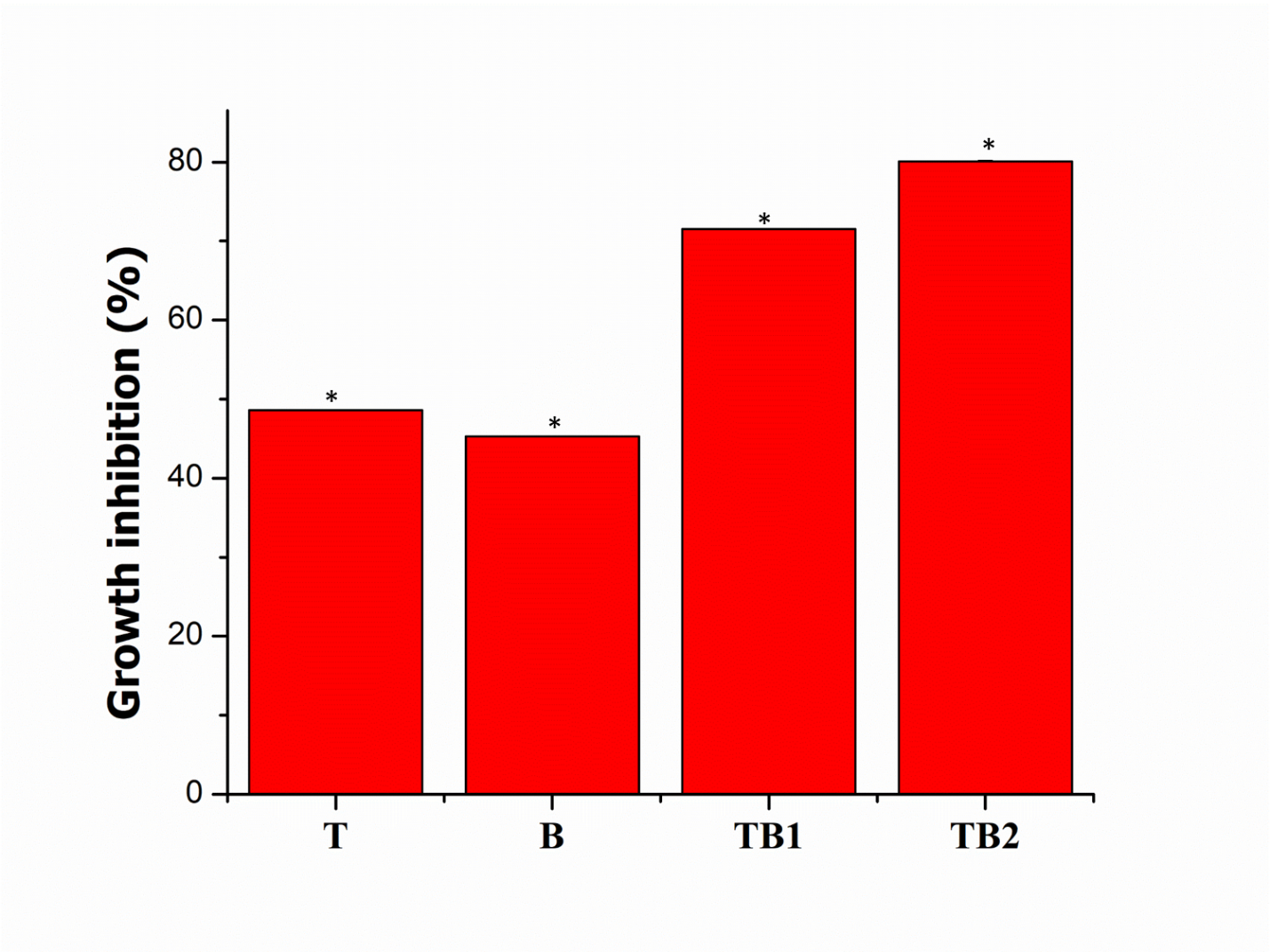


**Additional Table S1. Sequences of the primers used for the real-time PCR.**

| **Gene** | **Forward (5’-3’)** | **Reverse (5’-3’)** | **Reference** |
| --- | --- | --- | --- |
| ***VEL* 1** | CGAGGAGGGCAAGGACATTAC | GCAGGAACACCAGTCAGGATG | (Bazafkan et al., 2017) |
| ***TMK*** | CCAGCCCGACCATCATGTCTC | CGCATAATCTCTTGGTAAATCAGTTG | (Mukherjee et al., 2003) |
| ***GPR* 1** | TTGATCCAGACCTTCATGCCAGC | CATAAAAGGCCGCGACACGAA | (Brunner et al., 2008) |
| ***BLR* 1** | GGGATGACAGCCGAAC | TCAGCTCCCGCGTGAC | (Casas-Flores et al., 2004) |
| ***BLR* 2** | CATTGCGGCTGCTAGG | GTCCCTTTCGCCATTC |  |
| ***ENV1*** | GCCCTCTCGACTGCTCCGTC | CGACCCATGATCTCGGGGGC | (Bazafkan et al., 2017) |
| ***NAG* 1** | TCATTGGCGGTGAACTCG | TCGTCCCGACCACCAGAT | (Yang, 2017) |
| ***NAG* 2** | CAAGAGGACCATTGACGC | GCTTAGGCAGTGAAGGGAT |  |
| ***BGN13*** | GCAGCCCTCAACAAGGTG | AGAGCCAACGGGAACCAC |  |
| ***BGN* 16** | ATGAGTCTCCTGGAATGG | GTTGGGATGTAGTTGAGGT |  |
| ***EG1*** | CTACTTCGGCCCCGGAGA | GGTGATGCTGACGAGGTTG |  |
| ***ECH*** | AGTACCCTGCCGATGACA | TAGCCTGGAGCGTATTGC |  |
| ***PAP* A** | TGGACAGCATCAACACCG | ACTGAGCAGATGAGACCTGGCCGTAGT | (Chet et al., 2004) |
| ***PAP* B** | GACAGGACATTGCCTGGACT | GACTGGAC TCCTCCGTACCA |  |
| ***AF*** | ATGGAGCTTAAAGCACTCAG | TCAGCGCTGGAGAGTTAGC |  |
| ***NOX*** | CACCACCTGTTCATCCC | GTCAAATGGCGAGAATCC | (Montero-Barrientos et al., 2011) |
| ***CAT*** | ACTGCATTGTCCGTTTCT | AGTTGCCCTCCTCTGTG |  |
| ***ACC*** | GACCTGCTCCACCATCTTCC | CAGTGGAGTTGCCGACAAAG | (Viterbo et al., 2010) |
| ***NP1*** | GCGAATCAGAACAACAGCC | CATAGCCGTTCAGCCCATC | (Mukherjee & Kenerley, 2010) |
| ***NP2*** | CGTCCGTGGATATCCAGGC | GCCATCCGTATAGCCTGAC |  |
| ***NP3*** | CAAGACGCGTTTCACCTTCTTG | CGCTGTCCATTTGATCTCGC |  |
| ***Tri* 13** | CATGGATGCAATCTGGGCCATTGT | TGGCCGCCCATAATAAATCCGAGA |  |
| ***OMT*** | CACTGTTGCACAGGCTGTTCCATT | AAGTTGTACCACTGCTCCTCGGTT |  |
| ***PK1*** | AAGACAATCCAACCTATCGGGCCA | TCTGCAACATCACAAGGCACAACG |  |
| ***PK2*** | CGCGCAACTTCAACGCTCTTACAA | TCATAGGCACAAATACCTCCCGCA |  |
| ***18S rRNA*** | GGTGGAGTGATTTGTCTG | CTTACTAGGGATTCCTCG | (Tisch et al., 2011) |
| ***LoaP*** | GCAGGCTCTCTACTCAATCATAC | CAGCACATATCCCGGAAACA | (Goodson et al., 2017) |
| ***DfnA*** | CCCATTACCTATGCCGAAAGA | TCTTCGTTCCCTAATCTCATTCC |  |
| ***DfnG*** | ACAGCGAACTGACGGAATAC | GGCTGAGCTCAAGCTGATAA |  |
| ***DfnM*** | GGGCTGTCAGAAGAGTTTGT | ATTTCCGTAACCCGTCAGAAG |  |
| ***DlnA*** | TGATGCTGTTGCAGGACATAG | CTCGGAAGCCTCACTCATTAAC |  |
| ***DlnD*** | TTGGAAACGGAAGAAGGGATAG | ACGAACCGGAAGCGTATTT |  |
| ***DlnI*** | GAAGGCACCTTCTCACATCTT | ACGGCATGAGGATGAAACTC |  |
| ***16S rRNA*** | TCGCGGTTTCGCTGCCCTTT | AAGTCCCGCAACGAGCGCAA | (Cui et al., 2018) |
| **ACTIN** | CTCTCAGCACATTCCAGCAG | AGGAGGACGGCGATAACAG | (Saravanakumar et al., 2018) |
| **AOS** | ACCTGTTCACGGGCACCTAC | CGAGGAGCGAGGAGAAGTTG |  |
| **AOC** | CCCCTTCACCAACAAGGTGT | ACCGAGATGTGGCCGTAGTC |  |
| **ACS1** | GATGGTCTCGGATGATCACA | GTCGGGGGAAAACTGAAAAT |  |
| **PR1** | CTGGGTGTCCGAGAAGCAGT | CGGGTTGTAGCTGCAGATGAT |  |
| **PR10** | GTCATGCCGTTCAGCTTCAT | TGTTCTTGCACTCGACTTG |  |
| **PAL** | AAGAAGGTGAACGAGCTGGA | GTTGTCGTTCACGGAGTTGA |  |
| **PAL1** | TGTGCGTGCTTCTGCTGCTG | AGGGTGTTGATGCGCACGAG |  |
| **HPL** | ACTTCGGCTTCACCATCCTG | GTAGTAGCCCGGCCAGATGA |  |
| **LECTIN** | TCGTCGTCCTTGGAGAGCTT | CATCTGCCAAGTCCCCTTCT |  |
| **LIPASE** | CCAAGAGCCTCATCATCGTG | CGTGGTAGTGGTCCGTGTTG |  |
| **MFS** | CACTGTGGGCTGTGAGCAGT | GCAGGCCGAAATGTCTTGAT |  |
| **CYST2** | TGCCCTGCTCATACTGCTTG | GCGAGTTCCTGGAGGTGAAG |  |
| **PX5** | GGATTGATCCTGCGCTGAG | GACTCGAAGAGGCCCAGGTT |  |
| **CYST** | AGGGCTTGTTCGGTTAGGTG | TGCAGAATAAGGAGCCATGC |  |
| **THIOLASE** | TTCGCCCAAGTTTCAAGGAG | GCCGCATCTGCATATCCTCT |  |
| **TPS1** | TGCTGGCACCATGTTCTCTC | TCGTCCCACATCTCAACCAA |  |

References

Bazafkan, H., Dattenböck, C., Stappler, E., Beier, S., Schmoll, M. 2017. Interrelationships of VEL1 and ENV1 in light response and development in Trichoderma reesei. *PLOS ONE*, **12**(4), e0175946.

Brunner, K., Omann, M., Pucher, M.E., Delic, M., Lehner, S.M., Domnanich, P., Kratochwill, K., Druzhinina, I., Denk, D., Zeilinger, S. 2008. Trichoderma G protein-coupled receptors: functional characterisation of a cAMP receptor-like protein from Trichoderma atroviride. *Current genetics*, **54**(6), 283-299.

Casas-Flores, S., Rios-Momberg, M., Bibbins, M., Ponce-Noyola, P., Herrera-Estrella, A. 2004. BLR-1 and BLR-2, key regulatory elements of photoconidiation and mycelial growth in Trichoderma atroviride. *Microbiology*, **150**(Pt 11), 3561-9.

Chet, I., Harel, M., Viterbo, A. 2004. Isolation of two aspartyl proteases from Trichoderma asperellum expressed during colonization of cucumber roots☆. *FEMS Microbiology Letters*, **238**(1), 151-158.

Cui, W., Suo, F., Cheng, J., Han, L., Hao, W., Guo, J., Zhou, Z. 2018. Stepwise modifications of genetic parts reinforce the secretory production of nattokinase in Bacillus subtilis. *Microbial biotechnology*, **11**(5), 930-942.

Goodson, J.R., Klupt, S., Zhang, C., Straight, P., Winkler, W.C. 2017. LoaP is a broadly conserved antiterminator protein that regulates antibiotic gene clusters in Bacillus amyloliquefaciens. *Nat Microbiol*, **2**, 17003.

Montero-Barrientos, M., Hermosa, R., Cardoza, R.E., Gutiérrez, S., Monte, E. 2011. Functional analysis of the Trichoderma harzianum nox1 gene, encoding an NADPH oxidase, relates production of reactive oxygen species to specific biocontrol activity against Pythium ultimum. *Applied and environmental microbiology*, **77**(9), 3009-3016.

Mukherjee, P.K., Kenerley, C.M. 2010. Regulation of Morphogenesis and Biocontrol Properties in <em>Trichoderma virens</em> by a VELVET Protein, Vel1. *Applied and Environmental Microbiology*, **76**(7), 2345-2352.

Mukherjee, P.K., Latha, J., Hadar, R., Horwitz, B.A. 2003. TmkA, a mitogen-activated protein kinase of Trichoderma virens, is involved in biocontrol properties and repression of conidiation in the dark. *Eukaryotic cell*, **2**(3), 446-455.

Saravanakumar, K., Dou, K., Lu, Z., Wang, X., Li, Y., Chen, J. 2018. Enhanced biocontrol activity of cellulase from Trichoderma harzianum against Fusarium graminearum through activation of defense-related genes in maize. *Physiological and Molecular Plant Pathology*, **103**, 130-136.

Tisch, D., Kubicek, C.P., Schmoll, M. 2011. New insights into the mechanism of light modulated signaling by heterotrimeric G-proteins: ENVOY acts on gna1 and gna3 and adjusts cAMP levels in Trichoderma reesei (Hypocrea jecorina). *Fungal Genetics and Biology*, **48**(6), 631-640.

Viterbo, A., Landau, U., Kim, S., Chernin, L., Chet, I. 2010. Characterization of ACC deaminase from the biocontrol and plant growth-promoting agent Trichoderma asperellum T203. *FEMS Microbiology Letters*, **305**(1), 42-48.

Yang, P. 2017. The gene task1 is involved in morphological development, mycoparasitism and antibiosis of Trichoderma asperellum. *Biocontrol Science and Technology*, **27**(5), 620-635.
